# Occupational chemical exposures in pregnancy and fetal growth: evidence from the Born in Bradford Study

**DOI:** 10.5271/sjweh.3878

**Published:** 2020-07-01

**Authors:** Adeleh Shirangi, John Wright, Eve M Blair, Rosemary RC McEachan, Mark J Nieuwenhuijsen

**Affiliations:** Bradford Institute for Health Research, Bradford Teaching Hospitals, NHS Foundation Trust, Bradford UK; College of Arts, Business, Law, and Social Sciences, Murdoch University, Perth, WA, Australia; School of Population & Global Health, the University of Western Australia, Perth, WA, Australia; Telethon Kids Institute, Perth, WA, Australia; ISGlobal, Barcelona, Spain; Universitat Pompeu Fabra (UPF), Barcelona, Spain; CIBER Epidemiologíay Salud Pública (CIBERESP), Barcelona, Spain

**Keywords:** Key terms EDC, endrocrine disrupting chemical, epidemiology, maternal characteristic, percentage of optimal birth weight, prospective cohort, still birth

## Abstract

**Objectives:**

This prospective birth cohort study evaluated the effect of occupational exposure to endocrine disrupting chemicals (EDC) during pregnancy on inadequate fetal growth as measured by small-for-gestational age (SGA) and inadequate fetal growth measured by percentage of optimal birth weight (POBW). The study also identified the maternal characteristics associated with an increased risk of exposure to EDC.

**Methods:**

We studied 4142 pregnant women who were in paid employment during pregnancy and participated in a population-based, prospective 2007–2011 birth cohort study, the Born in Bradford Study, with an estimated participation of 80%. Job titles were coded at 26–28 weeks’ gestation at a 4-digit level according to 353 unit groups in the 2000 UK Standard Occupational Classification. They were then linked to expert judgment on exposure to each of ten EDC groups as assessed through a job exposure matrix (JEM). We performed generalized estimation equation modelling by a modified Poisson regression to assess the risk of POBW and SGA associated with an increased risk of chemical exposures.

**Results:**

The frequency of POBW<85 significantly increased for mothers exposed to pesticides [adjusted risk ratio (RR_adj_) 3.72, 95% confidence interval (CI) 1.40–9.91] and phthalates (RR_adj_ 3.71, 95% CI 1.62–8.51). There was a 5-fold increase risk of SGA for mothers exposed to pesticides (RR_adj_ 5.45, 95% CI 1.59–18.62). Veterinary nurses and horticultural trades were most frequently associated with exposure to pesticides while hairdressers, beauticians, and printing machine minders were associated with phthalates.

**Conclusion:**

Maternal occupational exposure to estimated concentrations of pesticides and phthalates is associated with impaired fetal growth.

Endocrine disrupting chemicals (EDC) are exogenous human-made substances that alter hormone regulation through interference with the endocrine system (1). They include many classes of chemicals such as pesticides, phthalates, polycyclic aromatic hydrocarbons (PAH), alkyl phenolic compounds (ALP), solvents, cytotoxic drugs, and anaesthetic gases. Global concerns have been raised in recent years over the potential adverse health effects of exposure to EDC (1–3). The endocrine system regulates many essential body functions such as growth, behavior, and reproduction through the controlled release of hormones (1, 4). The most sensitive windows of exposure to EDC are during fetal development and puberty (1). With an increasing number of women active in the labor force in both developed and developing countries, many will work during their reproductive years (5, 6) and likely be exposed to a variety of chemicals during pregnancy. Associations between prenatal exposure to EDC and a number of adverse pregnancy outcomes have been reported, including miscarriage (7), birth defects (8–12), stillbirth (13), small-for-gestational age (SGA) (14), impaired fetal growth (15, 16), low birthweight (LBW) (17), and preterm birth (PTB) (18). However, there are limited prospective birth cohort studies to evaluate this association and despite these investigations, evidence of such effects in humans is inconclusive, and many EDC have not yet been evaluated in epidemiological research (5).

Babies born with inadequate fetal growth are at increased risk of life-threatening health problems, as well as long-term complications and developmental delays (19–23). Inadequate fetal growth is an important predictor of perinatal morbidity and mortality, a potential risk factor for cognitive disability later in childhood and coronary heart disease and hypertension in adult life (17–21). Despite extensive research, the causes of these adverse birth outcomes are incompletely understood but factors such as sociodemographic and socioeconomic status; lifestyle; reproductive history; medical conditions, such as diabetes and hypertension during pregnancy; as well as occupational and environmental exposures may be relevant (24–27). Their association with several work-related risk factors is well established and has resulted in legislation, for example, considering exposure to specific chemicals, such as photoresistant solvents in the semiconductor industry or antineoplastic (cytotoxic) drugs in healthcare organizations, which have been declined over the past 20 years (11, 28). However, the scientific evidence is less consistent for many other EDC.

The primary objective of this study was to assess the effects of occupational exposures to estimated concentrations of EDC on the risk of SGA and inadequate fetal growth. The secondary objective was to identify the maternal characteristics associated with an increased risk of exposure to EDC.

## Methods

The Born in Bradford Study is a population-based, prospective, longitudinal, and multi-ethnic birth cohort study that recruited 12 453 pregnant women with 13 959 pregnancies during 2007–2011. With an estimated participation rate of 80%, the study monitors participants, their partners and off-spring until adulthood. Full details of the study methodology have been previously reported elsewhere (29, 30).

### Study design

**Figure 1:**
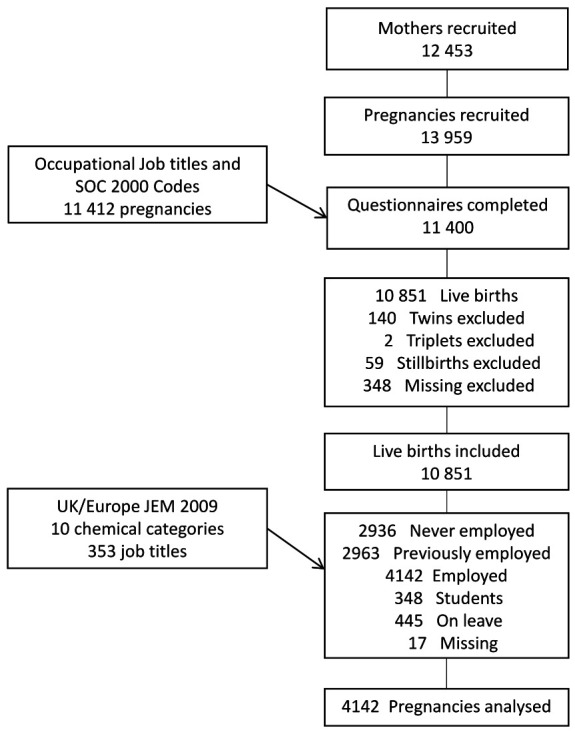
Flowchart of the steps in the selection of the study cohort.

Figure 1 shows the selection of the study cohort. Information about job description and working conditions was collected primarily through a mid-pregnancy questionnaire at about 26–28 weeks’ gestation. The questionnaire data was linked to maternity data and employment status for 11 400 pregnancies. We selected women who gave birth to a live-born singleton, were in paid employment during pregnancy, and enrolled in the Born in Bradford Study prenatally. Of 11 400 pregnancies, we excluded those with twins (N=140), triplets (N=2), stillbirths (N=59), and missing information on pregnancy outcome (N=348). Of the 10 851 remaining, we excluded pregnancies where the mother was: not employed during pregnancy (N=2963), Of the 10 851 remaining, we excluded pregnancies where the mother was: not employed during pregnancy (N= 2963), never employed (N=2936), a student (N=348), on sick leave (N=445), and missing information on the working situation (N=17). Therefore, 4142 (38%) of mothers in paid employment during their pregnancies were eligible for analysis.

The Bradford Research Ethics Committee provided ethics approval for the study (reference 06/Q1202/48).

### Working condition and occupational coding

Information concerning job title, type of business, self-employment, and the four main tasks performed at work were used to classify the jobs according to the UK Classification of Occupations (31). We coded the job titles at a 4-digit level according to 353 unit groups in the 2000 UK Standard Occupational Classification (31). The job titles were coded and validated through the Computer-Assisted Structure Coding Tool (Cascot) (32). The coded job titles subsequently linked to an updated UK job exposure matrix (JEM) for chemical exposure developed over the same period as this cohort study (33, 34).

### Exposure assessment

In 2009, Brouwers et al (34) developed this JEM, which considers the 353 job titles, adapted from the van Tongeren JEM of 2002 (33). Three occupational hygienists estimated the job-specific risk of exposure to each of ten chemicals groups: PAH, polychlorinated organic compounds, pesticides, phthalates, organic solvents, bisphenol A, ALP, brominated flame-retardants, metals, and a miscellaneous group: as unlikely (score=0), possible (score=1) or probable (score=2). In addition, broad and non-specific job titles were considered ’unclassifiable’. For this study, we collated the last two categories (possible and probable) into one indicating the occurrence of exposure to EDC was more likely than unlikely. No distinction was made between the various routes of exposure (inhalation, ingestion, or dermal). For many chemicals, most of the population experiences some level of exposure through diet or widely used consumer products. The JEM exposure score refers to the probability that the occupational exposure exceeds this background level.

### Measures of birth outcomes

Information about gestational age, gender, weight, length and head circumference at birth was obtained from medical records and hospital registries to allow the following variables to be created.

*Gestational age* was based on the actual and estimated date of delivery calculated by the physician or midwife from the dating scan (if available) or last menstrual period.

*SGA* was defined as a birth weight less than the 10th customized centile, using GROW software from 2013 (35, 36), www.gestation.net/cc/about.htm (37). The calculation of SGA was derived from maternal characteristics, birth weight and gestational age data recorded in the electronic maternity system at the Bradford Research Institute.

*Optimal birthweight* was estimated for each birth using a model derived from a population of singletons not exposed to any of the common risk factors for growth anomaly, with terms for infant gender, gestational duration, and maternal height and parity by a method validated and corrected for births before 30 weeks’ gestation (38, 39). Appropriateness of intrauterine growth is inferred from the ratio of the observed-to-optimal birth weight expressed as a percentage, percentage of optimal birth weight (POBW). The 10th percentile of weight in the original population was a POBW of 87% (38), therefore our criterion for inadequate fetal growth of POBW of <85 represents a slightly more stringent criterion than the 10th percentile, the criterion used for SGA. The method has been used in previously published studies (23, 40, 41).

### Maternal characteristics / confounder assessment

The following potentially confounding factors were also solicited with the mid-term questionnaire: mother’s sociodemographic, lifestyle, ethnicity, medical, and socioeconomic status [index of multiple deprivation 2010 (IMD)] as described in table 1. These characteristics are considered potential confounders for both aims of study investigation. We also considered each group of EDC as independent variables in the analysis to address the first aim of this study. Chemical exposures with numbers fewer than five records, which include polychlorinated organic compounds, bisphenol A, and flame-retardants, were not included in the analysis. As such, seven groups of chemicals were included in the analysis.

**Table 1:** Characteristics of 4142 pregnant women enrolled in Born in Bradford Study and their associations with crude risk of impaired fetal growth- [BMI=body mass index; EDC=endrocrine disrupting chemical; GCSE=general certificate of secondary education; IMD=index of multiple deprivation for Bradford; PAH= polycyclic aromatic hydrocarbons; POBW=percentage of optimal birth weight; SGA= small-for-gestational age.]

Characteristics	Number ^a^	%	SGA	Crude SGA risk	POBW<85	Crude POBW <85 risk
Total	4142	100	451	0.109	740	0.179
Ethnic origin						
British white	2482	59.92	188	0.076	323	0.135
South Asian	1188	28.68	228	0.191	352	0.307
Other	472	11.4	35	0.074	65	0.145
Age (years)						
≤35	3683	88.92	482	0.109	659	0.185
>35	459	11.88	49	0.107	81	0.183
Education (mother)						
<5 GCSE equivalents	315	7.62	29	0.092	70	0.235
5 GCSE equivalents	1120	27.11	142	0.126	210	0.195
A-level equivalent ^b^	819	19.82	86	0.105	148	0.184
>A level	1470	35.58	155	0.105	253	0.179
Other degrees	333	8.06	31	0.093	44	0.137
Unknown	75	1.82	8	0.107	14	0.192
Smoking (cigarettes)						
0	3492	84.37	354	0.101	575	0.171
1–5 per day	286	6.91	38	0.133	68	0.245
>5 per day	361	8.72	59	0.164	97	0.275
Other tobacco products						
No	4105	99.32	445	0.108	730	0.184
Yes	28	0.68	5	0.178	8	0.296
Drugs during pregnancy						
No	3528	99.07	379	0.107	633	0.183
Yes	33	0.93	7	0.212	11	0.354
Alcohol						
No /occasionally	2981	72.09	352	0.118	581	0.202
Yes	1154	27.91	98	0.085	158	0.142
Vitamin/iron						
No	2465	59.58	284	0.115	457	0.193
Yes	1672	40.42	166	0.099	282	0.173
Body mass index						
Normal	1087	29.99	165	0.152	263	0.249
Overweight	1606	39.88	166	0.103	268	0.174
Obese	1328	32.98	100	0.075	171	0.134
Underweight	6	0.15	3	0.5	4	0.666
Parity						
First child	2146	53.7	279	0.13	392	0.182
Second child & higher	1850	46.3	159	0.086	348	0.188
Baby sex						
Female	1973	47.63	231	0.117	377	0.197
Male	2169	52.37	220	0.101	363	0.175
Age at first period (years)						
12–13	1795	45.4	182	0.101	293	0.169
<12	598	15.12	73	0.122	116	0.201
>13	1561	39.48	176	0.113	294	0.195
Marital status						
Married/re-married	2489	60.15	319	0.128	504	0.209
Single	1537	37.14	128	0.0.83	219	0.147
Separated/divorced/widowed	112	2.71	4	0.036	17	0.165
IMD 2010 score						
1 (most deprived)	936	23.12	140	0.149	225	0.248
2	946	23.37	118	0.125	190	0.207
3	1010	24.95	92	0.091	164	0.17
4	875	21.62	70	0.08	115	0.136
5 (least deprived)	281	6.94	19	0.067	32	0.118
Finance						
Better off	1260	30.59	132	0.105	221	0.181
About the same	2027	49.21	225	0.111	368	0.189
Worse off	832	20.2	90	0.108	146	0.182
Job hours/week						
≤35	2204	53.37	257	0.116	428	0.202
>35	1926	46.63	193	0.1	310	0.166
Work type						
Most time/sitting	1678	47.86	172	0.102	276	0.168
Most time/standing	1387	39.56	172	0.124	280	0.205
Physical effort	441	12.58	38	0.086	81	0.187
Occupational group (Mother)						
Managers/seniors	320	7.74	29	0.09	45	0.146
Professionals	439	10.62	43	0.098	73	0.173
Associated professionals	676	16.35	63	0.093	100	0.153
Administration/secretarial	717	17.34	89	0.124	139	0.2
Skilled trades	45	10.09	4	0.089	8	0.182
Personal service	815	19.71	83	0.101	140	0.178
Customer service	500	12.72	66	0.123	97	0.186
Machine operatives	53	1.35	7	0.127	14	0.269
Elementary occupation	510	12.98	67	0.125	123	0.24
Gestational diabetes						
No	3878	93.78	434	0.112	782	0.188
Yes	257	6.22	17	0.066	38	0.152
Preclampsia						
No	3844	97	407	0.106	675	0.181
Yes	119	3	27	0.227	37	0.322
Pre-existing hypertension						
No	3926	98.82	427	0.108	702	0.185
Yes	47	1.18	8	0.17	13	0.289
Any EDC exposure						
No	3777	92.66	417	0.11	673	0.184
Yes	299	7.34	27	0.09	54	0.186
Exposure to PAH						
No	4012	98.43	438	0.109	716	0.185
Yes	64	1.57	6	0.094	11	0.18
Exposure to pesticides						
No	4069	99.83	442	0.108	724	0.185
Yes	7	0.17	2	0.285	3	0.429
Exposure to phthalates						
No	4000	98.14	440	0.11	717	0.185
Yes	76	1.86	4	0.053	10	0.135
Exposure to organic solvents						
No	3895	95.56	425	0.109	690	0.184
Yes	181	4.44	19	0.105	37	0.209
Exposure to akylphenolics						
No	3917	96.1	428	0.109	693	0.184
Yes	159	3.9	16	0.101	34	0.219
Exposure to metals						
No	4028	98.82	441	0.109	722	0.186
Yes	48	1.18	3	0.062	5	0.109
Exposure to miscellaneous						
No	4006	98.34	441	0.11	720	0.186
Yes	70	1.72	3	0.043	7	0.103

a Missing not included in the analysis.

b A-Levels, A–C equates to Level 2 attainment defined by the 2011 revision of the International Standard Classification of Education; ≥2 advanced levels or equivalent qualifications equate to Level 3 educational attainment.

### Strategy of statistical analysis

We used univariate and multivariate analyses with risk ratios (RR) and 95% CI (CI) generated using generalized estimation equation (GEE) modelling by a modified Poisson regression, with robust error variance (42, 43). Findings at P<0.05 were considered significant. The two crude and adjusted models estimated the risk of dependent variables with independent variables as shown in tables 2 and 3. All independent variables were categorical. For example, in table 2, all co-variables were screened by cross-tabulations, Chi^2^ test and also the Mantel-Haenzel adjusted odds ratio (OR) with separate SGA and POBW variables. If significant at P<0.2, the co-variables were entered into fully adjusted multivariate models for both SGA and POBW. Backwards-stepwise regression was used to simplify the models by sequentially removing non-significant variables that did not reduce how well the data fitted the models. Covariates were included in the multivariate model if the difference between the crude and adjusted RR was >10% for either outcome measure. For reasons of comparison and based on evidence from previous literature, maternal age, education, alcohol consumption, and job hours were included by default, independent of statistical significance. Interaction effects were examined for statistical significance. The analysis of POBW<85 was also stratified by ethnicity. All preceding calculations were made using the statistical program STATA (StataCorp, College Station, TX, USA).

**Table 2:** Univariate and multivariate relative risk (RR) estimation using generalized estimation model by a modified Poisson regression of the effects of maternal occupational exposures to endocrine disrupter chemicals (EDC) on risk of inadequate fetal growth in infants born in Bradford. **Bold denotes significance** (P<0.05). [CI=confidence interval; BMI=body mass index; GCSE=general certificate of secondary education; IMD=index of multiple deprivation for Bradford; PAH= polycyclic aromatic hydrocarbons; POBW=percentage of optimal birth weight; SGA= small-for-gestational age.]

Characteristics	SGA	P-value	SGA	P-value	POBW<85	P-value	POBW<85	P-value
			
	Crude RR (95% CI)		RR_adj_^a^ (95% CI)		Crude RR (95% CI)		RR_adj_^a^ (95% CI)	
Exposed to PAH ^b^	0.86 (0.40–1.84)	0.69	1.25 (0.53–2.94)	0.60	0.97 (0.57–1.67)	0.92	0.91 (0.46–1.80)	0.79
Exposed to pesticides ^b^	2.63 (0.81–8.51)	0.10	**5.45 (1.59–18.62)**	**0.00**	**2.32 (0.99–5.48)**	**0.05**	**3.72 (1.40–9.91)**	**0.00**
Exposed to phthalates ^b^	0.48 (0.18–1.24)	0.13	1.69 (0.34–8.41)	0.52	0.73 (0.41–1.29)	0.28	**3.71 (1.62–8.51)**	**0.00**
Exposed to organic solvents ^b^	0.96 (0.62–1.48)	0.86	1.08 (0.36–3.29)	0.88	1.13 (0.85–1.52)	0.39	0.89 (0.44–1.80)	0.74
Exposure to akylphenolics ^b^	0.92 (0.57–1.48)	0.73	1.62 (0.50–5.25)	0.42	1.19 (0.88–1.62)	0.25	1.48 (0.70–3.11)	0.31
Exposure to metals ^b^	0.57 (0.19–1.71)	0.31	0.58 (0.15–2.16)	0.42	0.58 (0.25–1.34)	0.2	0.49 (0.16–1.49)	0.21
Exposure to miscellaneous ^b^	0.39 (0.13–1.18)	0.09	0.23 (0.03–1.84)	0.17	0.55 (0.27–1.12)	0.09	0.18 (0.05–0.60)	0.00
Occupational group (mother)								
Managers/seniors	1.00		1.00		1.00		1.00	
Professionals	1.08 (0.69–1.69)	0.72	0.92 (0.50–1.69)	0.80	1.00 (0.81–1.24)	0.96	1.18 (0.75–1.86)	0.47
Associated professionals	1.02 (0.68–1.56)	0.86	1.10 (0.64–1.91)	0.72	1.01 (0.76–1.45)	0.91	1.35 (0.89–2.04)	0.15
Administration/secretarial	1.36 (0.92–2.03)	0.12	1.18 (0.69–2.01)	0.54	**1.21 (1.00–1.46)**	**0.04**	1.49 (0.98–2.24)	0.06
Skilled trades	0.98 (0.36–2.66)	0.97	0.63 (0.18–2.20)	0.48	1.07 (0.68–1.66)	0.76	0.99 (0.41–2.37)	0.99
Personal service	1.12 (0.75–1.68)	0.57	1.07 (0.61–1.87)	0.81	1.15 (0.95–1.39)	0.13	1.19 (0.78–1.82)	0.41
Customer service	1.36 (0.89–2.05)	0.14	1.05 (0.60–1.83)	0.85	1.15 (0.94–1.40)	0.15	1.18 (0.77–1.81)	0.44
Machine operatives	1.40 (0.65–3.05)	0.39	0.64 (0.22–1.86)	0.41	**1.51 (1.09–2.09)**	**0.01**	1.42 (0.66–3.06)	0.37
Elementary occupation	1.38 (0.92–2.10)	0.11	0.99 (0.55–1.78)	0.99	**1.31 (1.20–2.24)**	**0.00**	1.29 (0.84–2.00)	0.24
Work								
Most time/sitting	1.00		1.00		1.00		**1.00**	
Most time/standing	**1.21 (0.99–1.48)**	**0.05**	1.23 (0.96–1.58)	0.10	1.07 (0.98–1.18)	0.12	**1.25 (1.04–1.51)**	**0.01**
Physical effort	0.84 (0.60–1.17)	0.31	0.85 (0.57–1.28)	0.44	0.97 (0.83–1.12)	0.66	1.19 (0.91–1 .56)	0.18
Ethnic origin								
British Caucasian	1.00		1.00		1.00		**1.00**	
South Asian	**2.53 (2.11–3.03)**	**0.00**	**2.69 (1.94–3.73)**	**0.00**	**1.97 (1.81–2.13)**	**0.00**	**2.43 (1.91–3.09)**	**0.00**
Other	0.97 (0.69–1.38)	0.90	0.86 (0.52–1.41)	0.56	1.02 (0.87–1.19)	0.77	1.06 (0.76–1.47)	0.72
Age (years)								
≤35	1.00		1.00		1.00		1.00	
>35	0.97 (0.73–1.29)	0.87	1.64 (1.16–2.32)	0.00	0.90 (0.80–1.22)	0.14	1.17 (0.90–1.52)	0.25
Education (mother)								
<5 GCSE equivalents	1.00		1.00		1.00		1.00	
5 GCSE equivalents	1.37 (0.94–2.00)	0.10	1.51 (0.95–2.41)	0.08	**0.84 (0.72–0.97)**	**0.02**	0.96 (0.73–1.28)	0.82
A-level equivalent ^c^	1.14 (0.76–1.69)	0.53	1.11 (0.67–1.83)	0.66	**0.83 (0.71–0.97)**	**0.01**	0.78 (0.57–1.06)	0.11
>A level	1.14 (0.78–1.66)	0.49	1.24 (0.77–2.01)	0.37	**0.79 (0.68–0.92)**	**0.00**	0.88 (0.64–1.20)	0.41
Other degrees	1.00 (0.62–1.63)	0.97	1.29 (0.71–2.33)	0.41	**0.69 (0.56–0.85)**	**0.00**	0.70 (0.45–1.09)	0.11
Unknown/foreign	1.15 (0.55–2.42)	0.70	1.26 (0.51–3.12)	0.61	0.71 (0.49–1.02**)**	0.06	0.91 (0.50–1.67)	0.78
Smoking (cigarettes)								
0	1.00		1.00		1.00		1.00	
1–5 per day	1.31 (0.96–1.79)	0.09	**1.63 (1.13–2.35)**	**0.00**	**1.20 (1.04–1.39)**	**0.01**	**1.62 (1.26–2.09)**	**0.00**
>5 per day	**1.61 (1.25–2.08**)	**0.00**	**2.65 (1.89–3.71)**	**0.00**	**1.35 (1.20–1.53)**	**0.00**	**2.29 (1.79–2.92)**	**0.00**
BMI								
Normal	1.00		1.00		1.00		1.00	
Overweight	**0.68 (0.55–0.83)**	**0.00**	**0.76 (0.60–0.97)**	**0.02**	**0.75 (0.68–0.82)**	**0.00**	**0.76 (0.64–0.91)**	**0.00**
Obese	**0.49 (0.39–0.63)**	**0.00**	**0.60 (0.45–0.80)**	**0.00**	**0.60 (0.54–0.67)**	**0.00**	**0.58 (0.47–0.71)**	**0.00**
Underweight	**3.28 (1.45–7.41)**	**0.00**	3.29 (0.67–16.24)	0.14	**2.01 (1.01–2.29)**	**0.00**	1.65 (0.41–6.58)	0.48
IMD 2010 score								
1 (most deprived)	1.00		1.00		1.00		1.00	
2	**0.83 (0.66–1.04)**	0.11	0.82 (0.62–1.07)	0.15	**0.88 (0.79–0.98)**	**0.02**	**0.77 (0.64–0.94)**	**0.01**
3	**0.60 (0.47–0.78)**	**0.00**	0.83 (0.61–1.12)	0.23	**0.76 (0.68–0.85)**	**0.00**	**0.82 (0.66–1.01)**	**0.07**
4	**0.53 (0.41–0.70)**	**0.00**	0.81 (0.58–1.15)	0.25	**0.71 (0.63–0.80)**	**0.00**	**0.78 (0.60–0.99)**	**0.04**
5 (least deprived)	**0.45 (0.28–0.71)**	**0.00**	**0.44 (0.20–0.93)**	**0.03**	**0.55 (0.44–0.69)**	**0.00**	**0.58 (0.36–0.93)**	**0.02**

a Adjusted for all variables in the table and job hours during pregnancy (<35, ≥35): alcohol consumption (yes or no/occasionally), marital status (married/remarried, single, separated/divorced, widowed), parity (first child, second child & higher), gestational diabetes (no, yes), preeclampsia (no, yes), pre-exciting hypertension (no, yes), other tobacco (no, yes), and drugs during pregnancy (no, yes).

b Unexposed=reference group (not shown in the table). Each group of EDC is included in the analysis as an independent variable.

c A-Level- A–C equates to Level 2 attainment defined by the 2011 revision of the International Standard Classification of Education; ≥2 advanced Levels or equivalent qualifications equate to Level 3 educational attainment.

**Table 3:** Characteristics of 4142 pregnancies with live births by likelihood of maternal occupational exposure to endocrine disrupter chemicals (EDC) during pregnancy, Born in Bradford Study, 2007–2012. **Bold denotes significance** (P<0.05). [CI=confidence interval; BMI=body mass index; GCSE=general certificate of secondary education; IMD=index of multiple deprivation for Bradford; PAH= polycyclic aromatic hydrocarbons; POBW=percentage of optimal birth weight; RR=risk ratio; SGA= small-for-gestational age.]

Characteristics (N=4142)	Unlikely EDC exposure		Possible/ probable EDC exposure		Chi^2^	Pr	Univariate and multivariate analyses			P-value
		
	N	%	N	%			crude RR (95% CI)	P-value	RR_adj_(95%CI) ^a^	
Exposure to EDC										
Any exposure (total)	3777	92.66	299	7.34						
PAH	4012	98.43	64	1.57						
Polychloride organic compounds	4072	99.9	4	0.1						
Pesticides	4069	99.83	7	0.17						
Phthalates	4000	98.14	76	1.86						
Organic solvents	3895	95.56	181	4.44						
Bisphenol A	4076	98.62	0	0						
Alkylphenolics	3917	96.1	159	3.9						
Flame retardants	4074	99.95	2	0.05						
Metals	4028	98.82	48	1.18						
Miscellaneous	4006	98.34	70	1.72						
Occupational group (mother) ^b^										
Managers/seniors	272	7.2	8	2.68			1.00		1.00	
Professionals	435	11.52	3	1			1.00		1.00	
Associated professionals	715	18.93	0	0			1.00		1.00	
Administration/secretarial	535	14.16	0	0	489.4	<0.00	1.00		1.00	
Skilled trades	646	17.1	30	10.03			**7.94 (4.00–15.76)**	**<0.00**	**12.08 (4.95–29.43)**	**<0.00**
Personal service	34	0.9	11	3.68			**43.73 (20.01–95.58)**	**<0.00**	**52.92 (19.19–145.92)**	**<0.00**
Customer service	716	18.96	99	33.11			**21.73 (11.71–40.30)**	**<0.00**	**25.10 (10.50–60.02)**	**<0.00**
Machine operatives	29	0.77	17	5.69			**66.11 (32.83–133.13)**	**<0.00**	**80.40 (31.08–207.98)**	**<0.00**
Elementary occupation	395	10.46	131	43.81			**44.55 (24.26–81.82)**	**<0.00**	**56.40 (24.01–132.51)**	**<0.00**
Work type										
Most time/sitting	1593	49.77	48	10.05			1.00		1.00	
Most time/standing	1229	38.39	147	58.33	91.18	<0.00	**3.65 (2.65–5.01)**	**<0.00**	**1.83 (1.28–2.60)**	**<0.00**
Physical effort	379	11.84	57	22.62			**4.47 (3.08–6.46)**	**<0.00**	**1.64 (1.10–2.45)**	**<0.00**
Ethnic origin										
British Caucasian	2214	58.62	224	74.92			1.00		1.00	
South Asian	1139	30.16	35	11.71	46.18	<0.00	**0.32 (0.22–0.46)**	**<0.00**	**0.35 (0.22–0.56)**	**<0.00**
Other	424	11.23	40	13.38			0.94 (0.68–1.29)	<0.69	**0.65 (0.44–0.97)**	**<0.03**
Age (years)										
20–34	3089	81.78	242	80.94			1.00		1.00	
>34	553	14.64	36	12.04	9.93	<0.00	0.84 (0.59–1.18)	<0.32	1.04 (0.72–1.52)	<0.87
<20	135	3.57	21	7.02			**1.85 (1.22–2.81)**	**<0.00**	0.94 (0.61–1.44)	<0.78
Education (mother)										
<5 GCSE equivalents	262	6.95	50	16.78			1.00		1.00	
5 GCSE equivalents	1005	26.67	102	34.23			**0.57 (0.42–0.78)**	**<0.00**	0.79 (0.57–1.10)	<0.14
A-level equivalent ^c^	763	20.25	46	15.44	69.76	<0.00	**0.35 (0.24–0.52)**	**<0.00**	**0.58 (0.39–0.86)**	**<0.00**
>A level	1378	36.57	60	20.13			**0.26 (0.18–0.37)**	**<0.00**	0.77 (0.50–1.20)	<0.25
Other	296	7.86	31	10.4			**0.59 (0.39–0.90)**	**<0.01**	1.04 (0.66–1.62)	<0.89
Unknown	64	1.7	9	3.02			0.77 (0.39–1.49)	<0.43	0.72 (0.33–1.59)	<0.42
Smoking (cigarettes)										
0	3222	85.37	215	71.91			1.00		1.00	
1–5 per day	244	6.47	37	12.37	38.13	<0.00	**2.10 (1.51–2.91)**	**<0.00**	1.33 (0.96–1.84)	<0.08
>5 per day	308	8.16	47	15.72			**2.12 (1.57–2.84)**	**<0.00**	1.01 (0.73–1.40)	<0.94
Alcohol										
No/occasionally	2938	72.2	197	65.89			1.00		1.00	
Yes	1131	27.8	102	34.11	6.41	<0.01	**1.34 (1.06–1.69)**	**<0.01**	1.10 (0.86–1.42)	<0.39
BMI										
Normal	986	26.84	85	29.31			1.00		1.00	
Overweight	1462	39.8	113	38.97	1.69	<0.63	0.90 (0.68–1.18)	<0.46	1.00 (0.75–1.33)	<0.99
Obese	1220	33.22	91	31.38			0.87 (0.66–1.16)	<0.35	0.98 (0.73–1.32)	<0.91
Underweight	5	0.14	<5g	0.34			2.10 (0.34–12.71)	<0.41	4.56 (2.17–9.56)	<0.00
Parity										
First child	1946	53.45	161	55.52			1.00		1.00	
Second child & higher	1695	46.55	129	44.48	0.46	<0.49	0.92 (0.74–1.15)	<0.49	0.87 (0.68–1.10)	<0.28
Marital Status										
Married/remarried	2334	61.86	116	38.8			1.00		1.00	
Single	1336	35.41	174	58.19	63.35	<0.00	**2.43 (1.94–3.05)**	**<0.00**	1.22 (0.91–1.63)	<0.17
Separated/divorced/ widowed	103	2.73	9	3.01			1.69 (0.88-3.25)	<0.11	0.96 (0.47-1.92)	<0.92
IMD 2010 score BF										
1 (most deprived)	857	23.21	70	23.73			1.00		1.00	
2	861	23.32	77	26.1			1.08 (0.79–1.48)	<0.59	1.17 (0.85–1.60)	<0.29
3	907	24.57	82	27.8	6.9	<0.14	1.09 (0.80–1.49)	<0.55	**1.38 (1.00–1.90)**	**<0.04**
4	815	22.07	47	15.93			0.72 (0.50–1.03)	<0.07	1.05 (0.72–1.54)	<0.74
5 (least deprived)	252	6.83	19	6.44			0.92 (0.56–1.51)	<0.76	**1.79 (1.06–3.05)**	**<0.02**

a Adjusted for all variables in the table and job hours during pregnancy (<35, ≥35), alcohol consumption (drank alcohol during pregnancy (yes or no/occasionally), marital status (married/remarried, single, separated/divorced, widowed), parity (first child, second child & higher), gestational diabetes (no, yes), preeclampsia (no, yes), pre-exciting hypertension (no, yes), other tobacco (no, yes), and drugs during pregnancy (no, yes).

b The first four occupational groups combined and used as the reference group in univariate and multivariate analyses.

c A-Level- A–C equates to Level 2 attainment defined by the 2011 revision of the International Standard Classification of Education; ≥2 advanced levels or equivalent qualifications equate to Level 3 educational attainment.

## Results

Table 1 describes the maternal characteristics of the 4142 eligible pregnancies and gives crude risks of SGA (10.9%), and POBW<85 (17.9%). The cohort was multiethnic: ~60% were classified as Caucasian British, ~29% South Asian (Pakistani/Bangladesh/India), and ~11% other. The results from crude risks indicate that women were more likely to have babies with inadequate fetal growth as measured by both SGA and POBW<85, if they are from a South Asian ethnicity group, less educated, smoke, use drugs, live in most derived areas, or have preeclampsia or pre-existing hypertension.

Table 2 presents the univariate and multivariate estimates of RR for each of the two outcomes associated with statistically significant risk factors. In the multivariate analyses, all maternal characteristics and each category of seven groups of chemicals (those with numbers more than five records) were included except vitamin/iron supplementation and financial status, which were not statistically significant. There were no significant differences between occupational groups for either outcome. However, work involving standing most of time was associated with a 25% increased risk of having a baby with inadequate fetal growth.

### Effects of EDC on the risk of SGA

In multivariate analysis, the proportion of infants with SGA among women likely occupationally exposed to PAH, pesticides, phthalates, or ALP was statistically non-significantly higher than among women in the reference group, except for exposure to pesticides where it was 5 fold higher [adjusted RR (RRadj) 5.45, 95% CI 1.59–18.62]. No association was found between SGA and exposures to solvents, metals, and miscellaneous chemicals.

### Effects of EDC on the risk of POBW<85.

In multivariate analysis, the proportion of infants with POBW<85 among women likely occupationally exposed to pesticides (RRadj 3.72, 95% CI 1.40–9.91) and phthalates 3-fold (RRadj 3.71, 95% CI 1.62–8.51) was higher than that among the women in the reference group. Exposures to ALP was statistically non-significantly associated with increased risk of POBW<85. No association was found between POBW<85 and exposures to PAH, organic solvents, and metals. Exposure to the miscellaneous category had a protective effect.

The most frequently occurring occupations associated with exposure to pesticides with significant adverse effects on fetal growth were veterinary nurses, veterinary assistants, and horticultural trades. The main pesticides encountered were carbamates, organophosphates and pyrethroids. The most prevalent occupations associated with exposure to phthalates with significant adverse effects on fetal growth were hairdressers, beauticians and related occupations and printing machine minders. The phthalates most often encountered were DEHP, BBP, DBP, and DEP. The most prevalent occupations associated with exposure to ALP with significant effect on fetal growth were domestic cleaners, hairdressers and beauticians. The ALP most often encountered were alklylphenols and alkylphenolic ethoxylates.

Table 3 shows the distribution of pregnancy characteristics of 4142 stratified by likelihood of maternal occupational exposure to EDC during pregnancy. Almost 7.5% of the study cohort were classified as possibly or probably exposed to ≥1 of 10 classes of EDC. The most common encountered exposures were to organic solvents (4.5%) and ALP (4%) phthalates (1.9%), PAH (1.6%), metals (1.2%), and miscellaneous (1.7%). In general, women who were more likely to be exposed to EDC worked in skilled trades, personal service, elementary occupations, or as machine operators. In addition, their work involved prolonged standing or physical effort and they were more likely to be Causcasian British and less educated.

## Discussion

This study provides evidence that maternal occupational exposure during pregnancy to estimated concentrations of EDC – as classified by application of a JEM – is associated with significantly increased risk of impaired fetal growth. In particular, mothers exposed to pesticides were three to five times more likely to have an infant with suboptimal fetal growth as measured by POBW<85 and SGA respectively, and mothers exposed to phthalates were about three times more likely to have a baby with inadequate fetal growth measured by POBW<85. Maternal exposure to ALP was associated with a non-significant but increased risk of inadequate fetal growth as measured by SGA and POBW<85.

This study also demonstrated disproportionate exposure to EDC with personal risk factors in women. In general, women who were exposed to EDC were more likely to be Caucasian British, less educated, done work involving prolonged standing or physical effort and worked as skilled trades, personal service, machine operators and elementary occupations.

The study has several strengths primarily due to the large amount and detail of data available. The prospective design minimises recall bias, and selection bias was minimised by the 80% participation rate to the mid-pregnancy. Detailed information was collected about individual maternal characteristics and information obtained on chemical exposures through JEM, which enabled adjustment for potential confounders including adjustment for exposures to individual EDC in order to minimise the effect of possible confounding. The classification of EDC exposures was assessed independently and prior to knowledge of the outcomes by a recently updated JEM developed specifically to assess the association between occupational exposures to EDC and birth outcomes, thus information bias was largely eliminated. We were able to evaluate the effect of several EDC exposures on two different criteria for inadequate fetal growth.

In this study, we used POBW<85 as an indicator of inadequate intrauterine growth that is less dependent on the health of the reference population or the quality of their morphometric data than is percentile position on a birth weight distribution. The method uses optimal rather than expected growth as the standard and reports the ratio of the observed birth dimension to the optimal birth dimension rather than as being above or below a specified position of the population distribution of that dimension, avoiding the problems inherent in the use of percentile position.

The availability of job titles, the detailed information on work tasks or activities routinely performed, type of business and information about when the mother worked during her pregnancy has reduced non-differential misclassification of exposure and enabled a more accurate assessment of occupational exposures compared with studies with access only to job titles.

We validated the job title coding to get a more accurate code for each job title, so the potential for observer bias in the coding of occupational title status was minimised, improving the reliability and validity of the coded job titles. The risk for each category of EDC was estimated, rather than several EDC categories combined, allowing any differences between categories to be observed. Finally, JEM-based assessments of risk of exposures to chemical agents are more reliable than self-reported assessments (17).

A limitation was that individual exposures were not measured. However, Vandenberg et al (44), has reviewed the dose–response between endocrine disrupters and various health outcomes and the possibility of non-monotonic dose–responses. As stated by Vandenberg: “the endocrine system evolved to function when unbound physiologically active ligand (hormones) are present at extremely low doses”, “EDCs that mimic natural hormones have been proposed to follow the same rules and therefore have biological effects at low doses” (44, page 8). Another limitation is that the cells of JEM represent exposure probabilities, which are only a crude measure of exposure, so it needs to be interpreted with caution. Furthermore, the JEM does not consider specific chemicals but only broad groups thereof, and the mechanisms of action can vary between specific chemicals in a group (34). In this study, we reported the specific chemicals identified within each broad group of pesticides, phthalates and alkylphenolic compounds, but it was not possible to distinguish the role of each specific chemicals in their broad groups in the observed lower fetal growth rate. There is also a possibility of overlap between the categories of phthalates and alkyphenolic compounds among exposed mothers, so it was not possible to separate the specific role of each of these chemicals in inadequate fetal growth rate. Finally, too few mothers were exposed to some of EDC such as polychlorinated organic compounds, bisphenol A, and flame-retardants to allow evaluation of their associations with our outcomes. Exposure to the miscellaneous category had a protective effect, but due to small number of exposed people, the CI was very large, (95% CI 0.05–0.60) so the results should be interpreted with caution. Large CI were also observed in the results of occupational groups in table 3. There might be a risk of type 2 errors due to small samples of those exposed to some of EDC. However, for other EDC our results are compatible with those of previous similar studies (4, 16, 17), enhancing their credibility as well as our own.

This study introduces another approach (fetal growth measured by percentage of optimal birth weight) for defining adequacy of growth in assessing the effects of chemical exposures compared to other published studies. To our knowledge, this is the first study to show that the risk of inadequate fetal growth as measured by POBW<85 was significantly elevated following possible or probable maternal occupational exposures to one or more classes of EDC, particularly pesticides and phthalates. A recent study in the Generation R cohort using the same JEM found that occupational exposures to pesticides and phthalates during pregnancy were significantly associated with reduced placental weight and fetal length as estimated by ultrasounds and reduced fetal weight following mother’s exposures to phthalates and PAHs (16). Another study from Generation R cohort using the same JEM concluded that maternal occupational exposure to phthalates and pesticides was associated with adverse effects on fertility and pregnancy outcomes (17). A meta-analysis from a European large-scale prospective study using the same JEM also suggests that pregnant women classified as exposed to multiple EDC, including pesticides and phthalates, were at significantly higher risk of term low birth weight newborns in cohorts throughout Europe (4). Our finding in regard to non-significant association between POBW<85 as a measure of fetal growth and maternal exposure to ALP is in line with a recent study in the Generation R cohort using the same JEM in assessing occupational exposure to chemicals and fetal growth as measured by reduced fetal weight estimated from ultrasound-fetometry (16). Our finding about a significant association between maternal exposure to pesticides and SGA is supported by several studies (45–47). However, epidemiological studies on the effect of exposures to endocrine disrupters on pregnancy outcomes are not always consistent, warranting further research into this important topic. For example, the affected occupations associated with exposures to pesticides in this study were those classified as veterinary nurses and horticultural trades and, to phthalates, hairdressers, beauticians, and printing machine minders. The findings in the present study concerning exposure to pesticides and phthalates in hairdressers and agricultural activities and having infants with inadequate fetal growth is in accordance with previous findings (16, 17, 48–50). However, there were some studies in agricultural activities and among hairdressers that show conflicting results (51–54).

There is limited research evaluating the occupational and personal characteristics of women associated with occupational exposures to EDC. Evaluation of the influence of both occupational and personal risk factors (smoking, alcohol consumption, age, marital status, ethnic origin, education, BMI, socio-economic status) would help to improve our understanding of health hazards and develop a comprehensive preventive approach to achieve a longer, healthy working life. Unequal exposure to occupational exposure acting as EDC is an under-recognized risk factor that may play an important role in deriving the higher rates of adverse pregnancy outcomes among those affected populations.

Human development is most vulnerable to toxic substances and endocrine disruption in the early embryonic period. The restricted fetal growth associated with exposure to pesticides and phthalates during pregnancy is an important public health concern because restricted fetal growth is linked to adverse health later in life such as coronary heart disease, stroke, type 2 diabetes, and hypertension (55). It is therefore important to identify occupation-related risk factors for adverse pregnancy outcomes. Further larger studies are needed to confirm these findings and identify potential targets for prevention. Until then, precautionary prevention and control management of risks to health and safety at the workplace are recommended. In general, the precautions to be taken for the protection of the reproductive health of both women and men will not differ from the safeguarding of all workers. A national priority of supporting research on occupational causes of adverse reproductive outcomes recommended.

### Concluding remarks

Consistent with the results of other studies, this prospective birth cohort study provides evidence that occupational exposure to pesticides and phthalates may play a role in the etiology of inadequate fetal growth and SGA infants.
